# Expert consensus on multilevel implementation hypotheses to promote the uptake of youth care guidelines: a Delphi study

**DOI:** 10.1186/s12961-024-01167-x

**Published:** 2024-08-02

**Authors:** Eveline M. Dubbeldeman, Rianne M. J. J. van der Kleij, Evelyn A. Brakema, Mathilde R. Crone

**Affiliations:** 1 Department of Public Health and Primary Care, Leiden University Medical Center, Leiden, the Netherlands; 2 Department of Health Promotion, Maastricht University, Maastricht, the Netherlands

**Keywords:** Guidelines, Youth care, Implementation, Determinants, Strategies, Behaviour change technique, Delphi

## Abstract

**Background:**

The implementation of youth care guidelines remains a complex process. Several evidence–based frameworks aid the identification and specification of implementation determinants and strategies. However, the influence of specific strategies on certain determinants remains unclear. Therefore, we need to clarify which active ingredients of strategies, known as behaviour change techniques (BCTs), elicit behaviour change and improve implementation outcomes. With this knowledge, we are able to formulate evidence–based implementation hypotheses. An implementation hypothesis details how determinants and in turn, implementation outcomes might be influenced by specific implementation strategies and their BCTs. We aimed to identify (1) determinants relevant to the implementation of youth care guidelines and (2) feasible and potentially effective implementation hypotheses.

**Methods:**

A four–round online modified Delphi study was conducted. In the first round, experts rated the implementation determinants based on their relevance. Next, experts formulated implementation hypotheses by connecting BCTs and implementation strategies to determinants and were asked to provide a rationale for their choices. In round three, the experts reconsidered and finalised their hypotheses based on an anonymous overview of all formulated hypotheses, including rationales. Finally, the experts rated the implementation hypotheses based on their potential effectiveness and feasibility.

**Results:**

Fourteen experts completed the first, second, and third rounds, with 11 completed the final round. Guideline promotion, mandatory education, presence of an implementation leader, poor management support, knowledge regarding guideline use, and a lack of communication skills were reported as most relevant determinants. In total, 46 hypotheses were formulated, ranging from 6 to 9 per determinant. For each determinant, we provide an overview of the implementation hypotheses that were most commonly deemed feasible and potentially effective.

**Conclusion:**

This study offers valuable insights into youth care guideline implementation by systematically identifying relevant determinants and formulating hypotheses based on expert input. Determinants related to engagement and to knowledge and skills were found to be relevant to youth care guideline implementation. This study offers a set of hypotheses that could help organisations, policymakers, and professionals guide the implementation process of youth care guidelines to ultimately improve implementation outcomes. The effectiveness of these hypotheses in practice remains to be assessed.

**Supplementary Information:**

The online version contains supplementary material available at 10.1186/s12961-024-01167-x.

## Background

According to the Convention on the Rights of the Child [1], ‘All children must be able to grow up in a safe and healthy environment where there are plenty of opportunities, to develop as people and participate’. Unfortunately, not every child is given this opportunity, which raises worldwide concerns. In the Netherlands, the need for youth care has grown, with approximately 443 thousand children (10.0%) under 23 receiving youth care in 2019 [2], increasing to 467 thousand (10.6%) by 2022 [3]. Within the scope of this study, we defined youth care as the care provided for children and their families experiencing a variety of problems, such as parenting issues, adverse socioeconomic conditions, and psychosocial and stress–related problems [4, 5]. For example, it supports families in financial hardship with education, healthcare, and nutrition, provides counselling and psychiatric help for adolescents with psychosocial issues, and protects children from domestic violence, offering them safe places and helping families tackle underlying issues. Untreated, these issues can hinder a child's development and lead to severe consequences like school dropout [6], antisocial or delinquent behaviour [6–8], severe psychological disorders [6, 8–11], and child abuse [12]. Ensuring children receive adequate care is essential to safeguard their right to a secure and healthy upbringing, emphasized by the Convention on the Rights of the Child. This involves early detection of emotional, behavioural, and social problems, with professionals recognizing when to refer for specialist interventions. [13]. Early identification and treatment can mitigate problems later in life [14–16] and limit associated costs and risks to society [17, 18].

In Dutch youth care, several evidence–based guidelines and interventions (further referred to as youth care guidelines) exist including the Model Protocol for Child Abuse and Domestic Violence [19], the Youth Health Care Guideline for Psychosocial Problems [20], and the Kindcheck [21]. These guidelines aid the identification and/or management of child psychosocial problems, child abuse and neglect (CAN), and parenting problems and to assist parents with mental health problems. For example, the Model Protocol for Domestic Violence and Child Abuse provides clear steps for professionals dealing with signs of violence. It involves identifying signs, consulting colleagues and reporting centers as needed, engaging with the individuals involved, assessing the situation, and deciding the appropriate action. This structured approach equips professionals with a comprehensive framework to effectively respond to signs of violence, ensuring the well-being of those affected [19]. However, the availability of evidence–based guidelines does guarantee their optimal implementation in practice [22–26]. Konijnendijk [24] showed that, despite professionals’ familiarity with the content of the CAN guidelines and their positive perceptions, full adherence was low. Similarly, a study evaluating guidelines on positive parenting and family violence prevention showed that while about half of the professionals were familiar with the guidelines, only 14–16% applied them in practice [25]. The implementation of guidelines poses inherent challenges, especially within youth care. The interdisciplinary nature of the field, combined with the need to address sensitive topics with vulnerable families, heightens the complexity. Additionally, challenges arise from growing waiting lists, increasing administrative burdens, and persistent personnel shortages within youth care [27, 28]. Hence, research increasingly emphasises the implementation of guidelines and interventions. Various theoretical frameworks have been developed to guide and facilitate the implementation process, concentrating on determinants influencing implementation [29–31] and offering taxonomies for effective implementation strategies [32].

Studies have identified several determinants (i.e. barriers and facilitators) influencing the implementation of guidelines addressing CAN [24, 33–36], domestic violence during pregnancy [35, 37, 38], shaken baby syndrome [39], and childhood obesity [40]. Common determinants across these guidelines include issues related to time [24, 36–40] and knowledge [33–40]. Barriers specific to CAN and domestic violence during pregnancy guidelines include professionals' concerns about their own [38, 41–43] and/or patients’ safety [37, 38, 41, 42]. Understanding the determinants related to a problem is essential as they offer valuable insights into developing effective implementation strategies. By identifying the root causes and contributing factors driving the problem, we can develop strategies that directly address these underlying issues, leading to more effective and sustainable solutions. However, despite providing valuable insights for developing strategies to optimize implementation, some determinants are challenging to change in practice, such as limited time and financial resources [44]. To ensure an effective implementation process, it is recommended to focus on determinants that are (1) important for guideline implementation and (2) changeable in practice (i.e., adjustable determinants) [45]. Determinants considered important and changeable are further referred to as relevant determinants (Box 1). Implementation strategies, such as local consensus discussions and the use of opinion leaders, aim to address these determinants and optimize implementation [32]. However, the specific impact of these strategies on determinants and their potential role in either implementation success or failure remains unclear. For example, strategies like educational outreach visits, learning collaboratives, and educational materials are considered effective in skill development [46]. Yet, the success of these strategies is not solely dependent on their direct impact on determinants. Embedded within strategies, Behaviour Change Techniques (BCTs), are specific techniques designed to induce behaviour change, playing a crucial role in shaping implementation outcomes. Examples of BCTs include providing instructions on how to perform behaviours, action planning, and using prompts or cues [47]. Despite the acknowledged effectiveness of these strategies and BCTs, the optimal combination that significantly influences implementation outcomes remains unclear. There is a need for a comprehensive understanding of how strategies and BCTs collectively influence determinants and, consequently, impact implementation performance [48]. With this knowledge, we are able to formulate detailed, evidence–based strategies that effectively stimulate the implementation of youth care guidelines.

Box 1: Relevant determinants and implementation hypotheses We use the term relevant determinants to indicate those determinants that are (1) important for the implementation of youth care guidelines and (2) changeable in practice (i.e. adjustable to a large extent). An implementation hypothesis details how implementation determinants and implementation outcomes might be influenced by specific behavioral change techniques and implementation strategies.

In this study, we aimed to (1) identify the determinants most relevant to the implementation of youth care guidelines and (2) identify BCTs and combine them with implementation strategies to tackle barriers and strengthen facilitators. The present paper outlines a modified Delphi study designed to provide an overview of experts’ opinions on relevant determinants and feasible and potentially effective BCTs and implementation strategies. In selecting the Delphi study as our methodology, we recognize the importance of professionals’ expertise in the field of youth care implementation, providing a unique combination of theoretical knowledge, practical experience, and contextual awareness [49, 50]. The involvement of experts in a systematic and iterative process, allows us to draw upon their diverse perspectives, fostering a collaborative approach that is essential for addressing the challenges in the implementation of youth care guidelines. The theory–informed behaviour change (TIBC) method developed by French et al. [51] guided our Delphi study (Table 1). The TIBC method is a systematic approach for developing implementation interventions designed to change professionals’ behaviour based on theoretical frameworks, empirical evidence, and practical considerations. The executing of the second and third steps of the TIBC method formed the foundation of our Delphi study. This method significantly contributes to our study objectives by providing a structured and theory-based framework to (1) identify determinants and (2) intervention components (i.e., BCTs and implementation strategies) that might be effective in addressing these determinants. In line with the work by French et al. [46], we use the term ‘implementation hypotheses’ to detail how specific BCTs and implementation strategies might influence implementation determinants and implementation outcomes (Box 1).

**Table 1:** Steps for developing a theory–informed implementation intervention

Steps	Tasks	Application in Delphi study
STEP 1: Who needs to do what, differently?	• Identify the evidence–practice gap • Specify the behavior change needed to reduce the evidence–practice gap • Specify the health professional group whose behavior needs changing	Not applicable
STEP 2: Using a theoretical framework, which barriers and enablers need to be addressed?	• From the literature, and experience of the development team, select which theory(ies), or theoretical framework(s), are likely to inform the pathways of change • Use the chosen theory(ies), or framework, to identify the pathway(s) of change and the possible barriers and facilitators to that pathway • Use qualitative and/or quantitative methods to identify barriers and facilitators to behavior change	• Rating determinants on their importance and changeability. Based on previous research, a preselected list of determinants influencing youth care guideline implementation will be provided to experts [56]
STEP 3: Which intervention components (behavioral change techniques and implementation strategies) could overcome the modifiable barriers and enhance the facilitators?	• Use the chose theory, or framework, to identify potential behavior change techniques to overcome the barriers and enhance the facilitators • Identify evidence to inform the selection of potential behavior change techniques and implementation strategies • Identify what is likely to be feasible, locally relevant and acceptable and combine identified components into an acceptable intervention that can be delivered	• Aligning determinants with a feasible and potentially effective BCT [59]. To facilitate this process, experts will be given a preselected list of BCTs for each specific determinant, drawing from a recent synthesis of literature and an expert consensus study on links between determinants and BCTs [48, 60] • Include an implementation strategy from the ERIC project [32]. Experts will be provided with a preselected list of potential effective implementation strategies derived from prior literature[46]
STEP 4: How can behavior change be measured and understood	• Identify mediators of change to investigate the proposed pathways of change • Select appropriate outcome measures • Determine feasibility of outcomes to be measured	Not applicable

Table adapted from French, Green [51]

## Method

### Study design

This study employed a four–round Delphi method. Relevant determinants were identified in a single round, guided by the primary objective of quickly obtaining experts' opinions on the relevance of determinants influencing the implementation of youth care guidelines. The formulation of implementation hypotheses involved a more nuanced and iterative process, spanning three rounds. This multi-round design aimed to harness the collective expertise of the participants and attain a nuanced understanding of their perspective on implementation hypotheses. The Delphi method, known for gathering participants' opinions within their field of expertise, offers advantages such as expert anonymity, iteration with controlled feedback, and statistical aggregation of group responses. It minimizes irrelevant discussions and group pressure towards conformity [49, 50]. Because current information on implementation hypotheses is scarce, our goal was to obtain a hierarchical overview of experts' opinions on feasible and potentially effective hypotheses to influence implementation determinants, rather than striving for complete consensus. The Delphi study proved suitable for providing such information. Furthermore, the formulation of final hypotheses required multiple rounds and the use of embedded data, which is not feasible with a single questionnaire. We aimed to include experts in implementation research and practice-based experts, anticipating differing opinions from various perspectives. The Delphi method minimizes group effects, such as pressure and suppressed dissenting opinions, which might occur in focus groups.

**Fig. 1:**
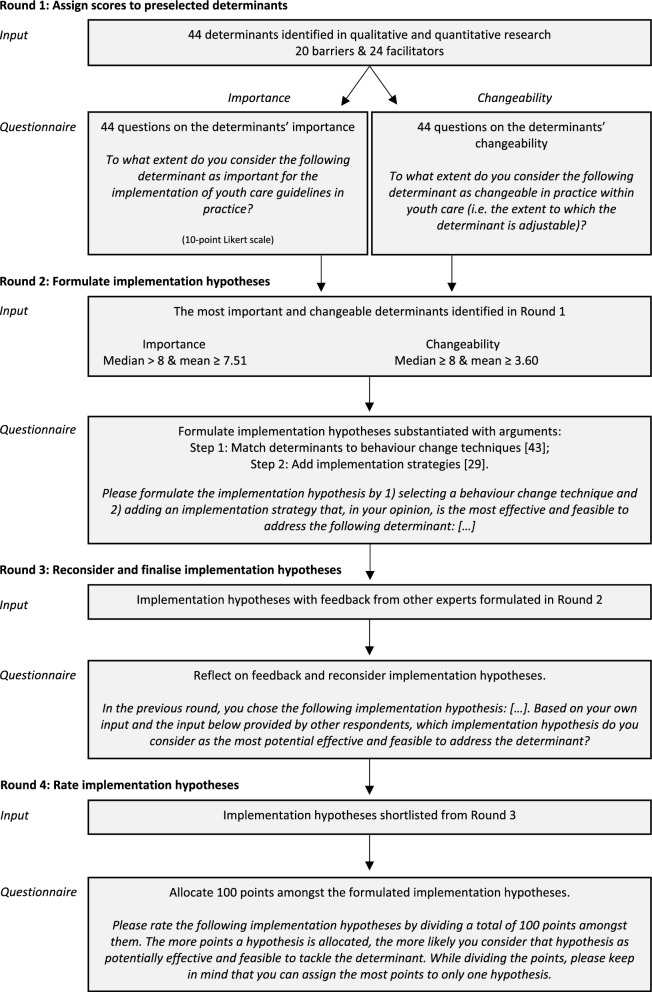
Overview of the four different rounds of this Delphi study

Online questionnaires were developed using Qualtrics [52], a web–based survey tool, and were administered over a four–month period (September–December 2020). Figure 1 provides an overview of the Delphi study, including example questions. Our reporting adheres to the Conducting and REporting of DElphi Studies (CREDES) recommendations [53] (Additional file 1). Research involving health professionals completing a questionnaire on the use of guidelines falls outside the scope of the Medical Research Involving Human Subjects Act (WMO) in the Netherlands [54], making ethical approval unnecessary. Nonetheless, participants were well-informed about the study's objectives, the commitment to participant anonymity during interactions, and the assurance of anonymity in publishing study outcomes, aligning with Delphi method principles. Complete anonymity to researchers posed challenges due to practical considerations, such as reminding participants to complete questionnaires and using embedded data between rounds, necessitating knowledge of participant identities. Despite these challenges, we prioritized ethical practices to ensure participants' voluntary and well-informed involvement.

### Preparation

The second step of the TIBC method involves identifying determinants that need addressing. In our approach, we conducted a modified Delphi study where the first round commenced with closed-ended questions on implementation determinants, deviating from the classical method that starts with open-ended questions [55]. This allowed us to present experts with a solid foundation of preselected determinants based on prior empirical research, minimizing their workload. Prior to the Delphi study, we conducted a systematic review to identify determinants influencing the implementation of youth care guidelines in general [56]. Additionally, non-published data on the implementation of a Dutch youth care guideline, Kindcheck, was utilized to identify determinants specific to this guideline. Based on these findings, we drafted a preliminary set of determinants, formulating them using the Consolidated Framework for Implementation Research (CFIR) developed by Damschroder [30]. The CFIR is an evidence–based framework that provides 39 constructs (i.e. determinants) arranged across five domains associated with effective implementation. Widely used in implementation research, the CFIR facilitates the practical application of results. In total, we identified 44 determinants, with 20 categorized as barriers and 24 as facilitators.

### Round 1 – Rank the preselected determinants

#### Participants and procedures

In the first round, our objective was to establish a ranking of determinants influencing the implementation of youth care guidelines based on experts' opinions regarding their relevance. We recruited experts in the field of youth care guidelines in the Netherlands through convenience sampling within the research network and snowball sampling. Potential participants received information about the study via mail and were invited to participate. Those who agreed received an email containing the link to the initial questionnaire. Reminders were sent to non-responders after one and two weeks.

#### Questionnaire

The questionnaire comprised questions on the importance and changeability of 44 determinants influencing youth care guideline implementation (a total of 88 questions). Experts were asked to rate each determinant on a 10-point Likert scale, ranging from 1 = not important to 10 = very important. The level of changeability was rated on a 5-point Likert scale, ranging from 1 = not changeable to 5 = very changeable. To minimize availability bias, we provided experts with determinant-specific results from the systematic review [56] and the Kindcheck implementation study. Availability bias is a mental shortcut leading individuals to draw conclusions based on readily available examples; if something is easily and quickly recalled, it may be perceived as important [57].

#### Analysis

To identify the determinants considered by the experts as the most relevant, we calculated median scores as indicators of the determinants’ importance and changeability. Following the approach by van Stralen et al. [58], determinants with a median score above 8 on a 10-point Likert scale were deemed important, while those with a median score of 4 on a 5-point Likert scale were considered changeable. In the first round, 19 determinants were identified as both important and changeable for the implementation of youth care guidelines. To avoid burdening the experts in the formulation of implementation hypotheses for too many determinants in the next round, determinants with a median score *above* 8 for importance were considered relevant. Mean scores were also considered, with determinants having a median score above 8 and a mean score of 7.51 or higher for importance, along with a median score of 4 or higher and a mean score of 3.60 or higher for changeability, being identified as the most relevant based on the grand mean of all determinants. These selected determinants served as inputs for the second round of this Delphi study.

### Round 2 – Formulate implementation hypotheses

In the second round, implementation hypotheses were formulated for each determinant resulting from Round 1. We used the term ‘implementation hypotheses’ to detail how the implementation determinants and implementation outcomes might be influenced by implementation strategies and their BCTs.

#### Questionnaire

Following the TIBC method, we asked the experts to match determinants with (1) a BCT formulated by Michie [59] and (2) an implementation strategy identified in the Expert Recommendations for Implementing Change (ERIC) project by Powell et al. [32].

In the first step, determinants were matched with BCTs. Experts were instructed to align each determinant with a feasible and potentially effective BCT [59]. To aid this process, a preselected list of effective BCTs for each specific determinant was provided, drawing from a recently conducted literature synthesis and expert consensus study on links between determinants and BCTs [48, 60]. This approach aligns with the TIBC method, which recommends reviewing relevant literature to identify BCTs with a positive impact on the determinants in question.

**Fig. 2:**
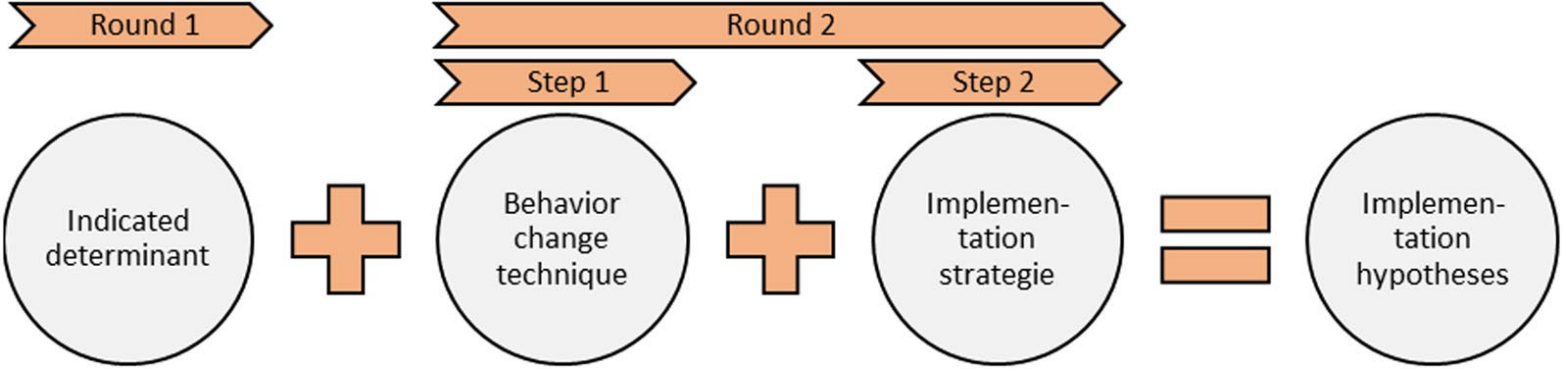
Process of implementation hypotheses formulation

The second step involved adding implementation strategies. Since the initial matches did not specify how BCTs could be practically delivered, experts were asked to include an implementation strategy from the ERIC project [32, 61]. The ERIC project offers a compilation of 73 strategies clustered into 9 categories (e.g., engage consumers, develop stakeholder interrelationships, train and educate stakeholders, etc.) to facilitate the implementation of innovations. This step led to the formulation of an implementation hypothesis. Initially, experts were provided with a preselected list of effective implementation strategies based on literature, as compiled by Waltz and colleagues [46]. After completing both steps, experts were prompted to elaborate on the rationale for their choices using an open-ended question. Figure 2 provides an overview of the process for formulating implementation hypotheses. To present the formulated hypotheses per determinant, frequency tables were employed.

### Round 3 – Reconsider and finalise the implementation hypotheses

The third round was conducted to reconsider and finalise the implementation hypotheses formulated in the second round.

#### Questionnaire

In the final round, participants were given an anonymous overview of hypotheses formulated by all experts for each determinant, along with the rationales for these hypotheses. Individual experts were then prompted to reconsider their initially chosen implementation hypotheses based on this collective overview. Experts had the option to either retain their own formulated implementation hypothesis or select one proposed by another expert. Frequency tables were utilized to list the formulated hypotheses for each determinant.

### Round 4 – Rate the implementation hypotheses

In the last round, the experts indicated which implementation hypotheses they considered most feasible and potentially effective in addressing specific determinants.

#### Questionnaire

Our research aimed to establish a hierarchical order for potentially effective implementation hypotheses. employing a ranking-type Delphi method with the fixed-sum approach [62, 63]. Experts were asked to rate implementation hypotheses for each determinant by allocating a total of 100 points (either in full or in part) to the hypotheses formulated in Round 3. This method facilitated data analysis through simple parametric tests, such as average points and standard deviation. Experts had flexibility in distributing points, with the only exception being that they could allocate the most points to only one hypothesis. The web-based survey tool ensured that experts could proceed to the next question only if they had allocated exactly 100 points. Data analysis was conducted using IBM SPSS Statistics version 24.

## Results

### Expert panel

In total, we approached 25 experts, and 19 expressed interest in participating. Fourteen experts completed the first, second, and third rounds (56% response rate), and 11 participated in the final round. All experts were Dutch and were experienced in guideline implementation: three had practice-based experience, and eleven had research-based experience in (youth care) guideline implementation (Additional file 2).

### Round 1

The results from Round 1 are presented in Additional file 3. The determinants considered most important to youth care guideline implementation by experts included poor management support (median = 9.00; mean = 8.64), a lack of communication skills (median = 9.00; mean = 8.43), the presence of a motivated implementation leader (median = 9.00; mean = 8.86), and professionals’ belief in positive outcomes for the child (median = 9.00; mean = 8.64). Perceived as least important were professionals’ fear of making a false identification (median = 6.00; mean = 6.07), a lack of equipment (median = 6.50; mean = 6.21), low confidence in follow–up care by external organisations (median = 6.00; mean = 6.29), and the opportunity for professionals to make an anonymous call to external organisations (median = 6.00; mean = 5.57).

Regarding changeability, the determinants indicated as most changeable included poor procedural clarity (median = 4.50; median = 4.36), mandatory education (median = 4.50; mean = 4.43), guideline promotion (median = 4.50; mean = 4.36), and the presence of a motivated implementation leader (median = 4.50; mean = 4.29). The determinants identified by the experts as least changeable were a lack of time (median = 2.00; mean = 2.64), poor congruence in the current workflow (median = 2.00; mean = 2.71), professionals' fear of harming the relationship with their client (median = 2.50; mean = 2.86), and availability of time (median = 2.00; mean = 2.57).

Six determinants were identified as the most relevant for youth care guideline implementation, serving as the basis for the second round (Table 2). Organizing these determinants based on their alignment within CFIR constructs, they were categorized into two groups: (1) engagement (i.e., guideline promotion, mandatory education, presence of a motivated implementation leader, and poor management support) and (2) knowledge and skills (i.e., guideline knowledge and poor communication skills).

**Table 2:** Determinants considered by expert as most relevant for youth care guideline implementation (n = 14)

Cat	Determinant	Importance		Changeability		CFIR construct (domain) [30]	Description of CFIR construct [30]
		Mean	Median	Mean	Median		
Engagement	Promotion of guideline use	8.50	8.50	4.36	4.50	Engaging (Process)	Attracting and involving appropriate individuals in the implementation and use of the intervention through a combined strategy of social marketing, education, role modeling, training, and other similar activities
	Mandatory education	8.07	8.50	4.43	4.50	Engaging (Process)	Attracting and involving appropriate individuals in the implementation and use of the intervention through a combined strategy of social marketing, education, role modeling, training, and other similar activities
	Presence of a motivated implementation leader	8.86	9.00	4.29	4.50	Engaging (Process)	Individuals from within the organization who have been formally appointed with responsibility for implementing an intervention as coordinator, project manager, team leader, or other similar role
	Poor management support	8.64	9.00	3.64	4.00	Leadership engagement (Inner setting)	Commitment, involvement, and accountability of managers with the implementation
Knowledge & skills	Knowledge regarding use of the guideline	8.29	8.50	3.79	4.00	Knowledge & beliefs about the innovation (Characteristics of individuals)	Individuals’ attitudes toward and value placed on the intervention as well as familiarity with facts, truths, and principles related to the intervention
	Lack of communication skills	8.43	9.00	3.79	4.00	Other personal attributes (Characteristics of individuals)	A broad construct to include other personal traits such as tolerance of ambiguity, intellectual ability, motivation, values, competence, capacity, and learning style

*Cat* category, *CFIR* Consolidated Framework for Implementation Research

### Rounds 2 and 3

In Round 2, a total of 60 different implementation hypotheses were formulated, with each determinant having between 9 to 11 different hypotheses. After the experts reevaluated their hypotheses based on anonymous feedback from other experts, a total of 46 hypotheses were formulated in Round 3, ranging from 6 to 9 different hypotheses per determinant (Additional file 3). Table 3 offers an overview of the two main implementation hypotheses most frequently considered by experts as feasible and potentially effective.

**Table 3:** Top 2 of implementation hypotheses mostly considered by experts as effective and feasible (n = 14)

	Implementation hypotheses		n (%)		Category
	Behavior change technique	Implementation strategy	R2	R3	Implementation strategy^a^
Engagement	*Promotion of guideline use*				
	Habit formation	Use advisory boards and workgroups	3 (21.4)	6 (45.8)	Develop stakeholder interrelationships
		Conduct educational meetings	2 (14.3)	2 (14.3)	Train and educate stakeholders
	Prompts/cues	Identify and prepare champions	2 (14.3)	2 (14.3)	Develop stakeholder interrelationships
	*Mandatory education*				
	Action planning	Create a learning collaborative	2 (14.3)	2 (14.3)	Train and educate stakeholders
		Conduct local needs assessment	3 (21.4)	4 (28.5)	Use evaluative and iterative strategies
		Use advisory boards and workgroups	1 (7.1)	2 (14.3)	Develop stakeholder interrelationships
	Restructuring the physical environment	Create a learning collaborative	2 (14.3)	2 (14.3)	Train and educate stakeholders
	*Presence of an implementation leader*				
	Social support (practical)	Provide ongoing consultation	2 (14.3)	3 (21.4)	Train and educate stakeholders
	Restructuring the social environment	Recruit, designate and train for leadership	2 (14.3)	3 (21.4)	Develop stakeholder interrelationships
	*Poor management support*				
	Social support (practical)	Conduct local consensus discussions	3 (21.4)	5 (35.7)	Develop stakeholder interrelationships
		Obtain formal commitments	2 (14.3)	2 (14.3)	Develop stakeholder interrelationships
Knowledge & skills	*Knowledge regarding the use of the guideline*				
	Feedback on behavior	Create a learning collaborative	2 (14.3)	3 (21.4)	Train and education stakeholders
	Instruction how to perform a behavior	Conduct educational meetings	3 (21.4)	4 (28.5)	Train and education stakeholders
	*Lack of communication skills*				
	Behavioral practice/rehearsal	Conduct educational outreach visits	3 (21.4)	2 (14.3)	Train and education stakeholders
		Conduct ongoing training	4 (28.5)	8 (57.1)	Train and education stakeholders

*R1* Round1, *R2* Round 2; ^a^Categories based by Waltz et al. [61]

#### Engagement

To facilitate change in determinants related to engagement, the BCT practical support was predominantly considered feasible and potentially effective (*n* = 10, 26.8%, Additional file 3). Additionally, various strategies for developing stakeholder interrelationships, such as using advisory boards and workgroups, obtaining formal commitments, and involving executive boards, were widely viewed as feasible and potentially effective in addressing determinants in practice (*n* = 35, 62.5%).

#### Knowledge and skills

Providing instructions on how to perform a behaviour was largely considered a feasible and potentially effective BCT to address knowledge about guideline use (*n* = 6, 42.9%). In tackling the lack of communication skills, behavioural practice/rehearsal was deemed feasible and potentially effective by the majority (*n* = 11, 78.6%). Various implementation strategies for training and educating stakeholders were most frequently considered feasible and potentially effective in addressing knowledge and skills in practice (*n* = 26, 92.9%).

### Round 4

**Fig. 3:**
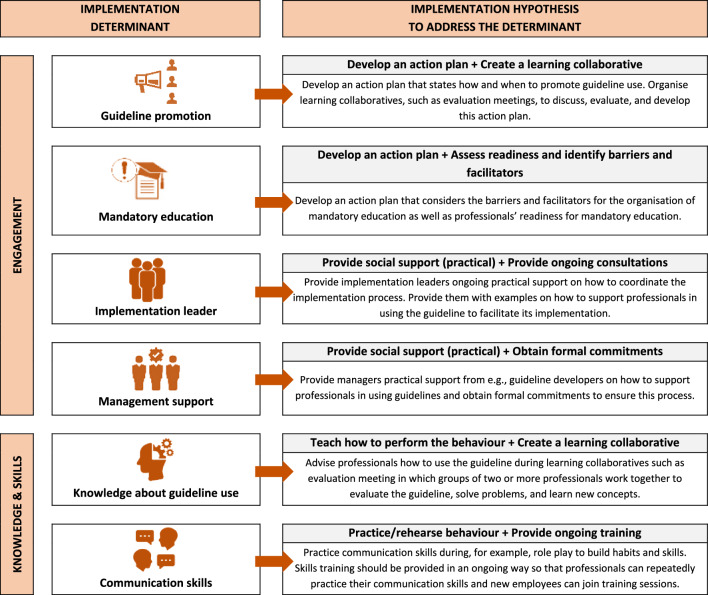
Overview of the determinants considered by the experts the most important and changeable for the implementation of youth care guidelines and possible strategies to address these determinants

We compiled a list of hypotheses ranked by the average points each implementation hypothesis received (Table 4). Figure 3 provides a simplified overview of the potential implementation hypotheses for each determinant based on the highest average points regarding their feasibility and potential effectiveness, as evaluated by the experts.

**Table 4:** Summary of results of round four (n = 11)

Implementation hypotheses			Expert											Tot	Mean (SD)	Rank	Category
Behavior change technique		Implementation strategy	2	4	5	6	7	8	9	10	11	12	14				Implementation strategy^a^
Engagemen**t**	*Promotion of guideline use*																
	Habit formation	Use advisory boards and workgroups	0	0	0	10	20	0	0	10	0	10	0	50	4.55 (6.88)	7	Develop stakeholder interrelationships
		Conduct educational meetings	10	0	20	20	20	0	60	9	0	35	0	174	15.82 (18.58)	3	Train and educate stakeholders
	Prompts/cues	Identify and prepare champions	35	20	0	10	30	0	0	20	30	0	0	145	13.18 (14.19)	5	Develop stakeholder interrelationships
		Conduct local needs assessment	30	20	20	10	20	0	0	17	0	0	50	167	15.18 (15.66)	4	Use evaluative and iterative strategies
		Conduct educational meetings	20	10	0	20	10	50	40	15	0	35	0	200	18.18 (17.07)	2	Train and educate stakeholders
	Action planning	Create a learning collaborative	5	30	60	20	0	30	0	11	50	20	40	266	24.18 (20.03)	1	Train and educate stakeholders
		Identify and prepare champions	0	20	0	10	0	20	0	18	20	0	10	98	8.91 (9.22)	6	Develop stakeholder interrelationships
	*Mandatory education*																
	Action planning	Create a learning collaborative	5	0	0	20	20	0	70	8	30	40	0	193	17.55 (22.14)	3	Train and educate stakeholders
		Conduct local needs assessment	30	70	0	20	40	0	0	12	0	0	50	222	20.18 (24.42)	2	Use evaluative and iterative strategies
		Use advisory boards and workgroups	30	0	30	10	20	0	0	14	0	0	0	104	9.45 (12.30)	5	Develop stakeholder interrelationships
		Assess for readiness and identify B&F	35	30	0	20	20	0	20	15	30	15	50	235	21.36 (14.68)	1	Use evaluative and iterative strategies
	Restructuring the physical environment	Assess for readiness and identify B&F	0	0	40	10	0	0	0	16	0	15	0	81	7.36 (12.59)	7	Use evaluative and iterative strategies
		Inform local opinion leaders	0	0	0	10	0	0	0	11	0	0	0	21	1.90 (4.25)	8	Develop stakeholder interrelationships
		Create a learning collaborative	0	0	30	10	0	70	10	9	0	30	0	159	14.45 (21.64)	4	Train and educate stakeholders
		Use advisory boards and workgroups	0	0	0	0	0	30	0	15	40	0	0	85	7.73 (14.38)	6	Develop stakeholder interrelationships
	*Presence of a motivated implementation leader*																
	Social support (practical)	Provide ongoing consultation	35	10	10	20	10	30	70	15	50	0	0	250	22.73 (21.84)	1	Train and educate stakeholders
		Identify and prepare champions	40	20	0	20	10	0	0	19	0	0	60	169	15.36 (19.66)	3	Develop stakeholder interrelationships
		Inform local opinion leaders	0	0	0	10	10	0	0	18	0	0	0	38	3.45 (6.27)	8	Develop stakeholder interrelationships
	Social support (unspecified)	Inform local opinion leaders	0	20	0	10	10	0	0	7	0	25	0	72	6.55 (8.96)	7	Develop stakeholder interrelationships
		Assess for readiness and identify B&F	0	20	30	10	20	0	0	8	0	0	40	128	11.64 (14.05)	4	Use evaluative and iterative strategies
		Identify and prepare champions	0	30	0	10	40	0	0	16	0	0	0	96	8.73 (14.21)	6	Develop stakeholder interrelationships
	Social comparison	Recruit, designate and train for leadership	25	0	50	10	0	70	0	7	20	40	0	222	20.18 (23.86)	2	Develop stakeholder interrelationships
	Restructuring the social environment	Recruit, designate and train for leadership	0	0	10	10	0	0	30	10	30	35	0	125	11.36 (13.80)	5	Develop stakeholder interrelationships
	*Poor management support*																
	Social support (practical)	Conduct local consensus discussions	30	0	30	20	10	30	10	9	10	0	15	164	14.91 (11.23)	2	Develop stakeholder interrelationships
		Obtain formal commitments	20	60	0	10	20	20	70	7	0	20	50	277	25.18 (24.03)	1	Develop stakeholder interrelationships
		Involve executive boards	20	40	0	20	10	30	0	11	0	0	0	131	11.91 (14.00)	5	Develop stakeholder interrelationships
		Recruit, designate and train for leadership	20	0	30	10	10	20	0	14	0	50	0	154	14.00 (15.62)	3	Develop stakeholder interrelationships
	Restructuring the social environment	Develop a formal implementation blueprint	0	0	0	10	0	0	20	7	0	0	0	37	3.36 (6.52)	9	Use evaluative and iterative strategies
		Obtain formal commitments	0	0	30	10	30	0	0	8	0	20	35	133	12.09 (14.07)	4	Develop stakeholder interrelationships
	Social comparison	Involve executive boards	0	0	0	10	0	0	0	10	30	10	0	60	5.45 (9.34)	7	Develop stakeholder interrelationships
		Identify and prepare champions	0	0	10	10	20	0	0	22	40	0	0	102	9.27 (13.18)	6	Develop stakeholder interrelationships
	Social rewards	Alter incentive/allowance structures	10	0	0	0	0	0	0	12	20	0	0	42	3.82 (6.95)	8	Utilize financial strategies
Knowledge & skills	*Knowledge regarding the use of the guideline*																
	Feedback on behavior	Conduct educational meetings	24	0	10	10	0	0	10	9	30	0	0	93	8.45 (10.35)	5	Train and educate stakeholders
		Create a learning collaborative	30	60	40	10	0	0	10	8	0	40	0	198	18.00 (20.98)	3	Train and educate stakeholders
	Instruction on how to perform a behavior	Conduct educational meetings	20	0	0	20	50	30	60	12	30	0	0	222	20.18 (20.89)	2	Train and educate stakeholders
		Create a learning collaborative	26	40	30	20	0	60	20	11	0	35	0	242	22.00 (18.97)	1	Train and educate stakeholders
	Information about antecedents	Distribute educational materials	0	0	0	10	20	10	0	7	0	0	0	47	4.27 (6.69)	8	Train and educate stakeholders
		Identify and prepare champions	0	0	20	10	10	0	0	20	0	0	0	60	5.45 (8.20)	7	Develop stakeholder interrelationships
	Information about health consequences	Conduct educational meetings	0	0	0	10	20	0	0	15	40	25	50	160	14.55 (17.67)	4	Train and educate stakeholders
		Conduct educational outreach visits	0	0	0	10	0	0	0	18	0	0	50	78	7.09 (15.40)	6	Train and educate stakeholders
	*Poor communication skills*																
	Behavioral practice/rehearsal	Conduct educational outreach visits	35	20	30	10	30	10	40	17	0	10	20	222	20.18 (12.28)	2	Train and educate stakeholders
		Conduct ongoing training	25	0	10	20	0	50	15	26	40	35	25	246	22.36 (15.73)	1	Train and educate stakeholders
		Create a learning collaborative	5	60	10	20	10	30	10	23	30	5	5	208	18.91 (16.65)	3	Train and educate stakeholders
		Assess for readiness and identify B&F	0	20	30	20	30	10	5	20	10	10	20	175	15.91 (9.70)	4	Use evaluative and iterative strategies
	Demonstration of the behavior	Conduct ongoing training	25	0	20	20	0	0	10	10	20	25	25	155	14.09 (10.44)	5	Train and educate stakeholders
		Conduct educational meetings	10	0	0	10	30	0	20	4	0	15	5	94	8.55 (9.81)	6	Train and educate stakeholders

*Tot* total points allocated to hypotheses, *SD* standard deviation, *B&F* barriers & facilitators

^a^Categories based by Waltz et al., 2015 [61]

#### Engagement

Across the hypotheses, the highest number of points were allocated to the BCTs practical support and action planning (1183, 26.9% and 1118, 25.4%, respectively) and diverse implementation strategies regarding the development of stakeholder interrelationships (2246, 51.0%). Specifically, for guideline promotion, the hypotheses ‘detailed action plan to promote guideline use that should be discussed and revised during collaborative learning sessions’ received the most points (266, 24.2%).

*“This takes time and congruence with all other guidelines. Prevent single mindedness (living one guideline....).Make teams responsible for their aptness to understand and practice all guidelines in an integrated and continuous way!” [respondent 8]*

For organizing mandatory education, experts allocated the highest number of points to the following hypothesis: ‘detailed action plan in which the necessities, barriers, and facilitators for the organization of mandatory education, as well as professionals’ readiness to mandatory education, are taken into account’ (235, 21.4%).

*“Involve the students in the planning and create time and space when necessary. Send a tailor out don't use a one size fits all approach. That does not motivate and does not work!” [respondent 8]*

The hypothesis ‘provide implementation leaders ongoing consultations on how to perform their tasks and keep them motivated’ received the highest number of points for its potential to enhance the presence of a motivated implementation leader (250, 22.7%).

*“My experience is that a the effort and the energy of the implantation leader is a reflection for those who have to implement, like a mirror” [respondent 2]*

‘Obtaining formal commitments from, for example, guideline developers, that states they will provide management practical support on how to support professionals in using guidelines’ received the most points to address poor management support (277, 25.2%).

*“Commitment written down may help professionals to talk to their managers regarding their responsibilities.” [respondent 5]*

#### Knowledge and skills

Across hypotheses, the experts allocated the highest number of points to the BCTs behavioural practice/rehearsal and instructions on how to perform a behaviour (851, 38.7% and 464, 21.7%, respectively) and various implementation strategies regarding the training and education of stakeholders (1965, 89.3%). More specifically, the hypothesis ‘providing instructions to professionals during collaborative learning sessions’ was mostly considered feasible and potentially effective in facilitating knowledge transfer regarding guideline use (242, 22.0%).

*“If specific knowledge is required it is needed that people can actually obtain that knowledge and so I chose the technique that deals with that knowledge, by creating a learning collaborative you have a structured and sustainable way of improving people's knowledge. You can share materials etc. within that group to foster learning.” [respondent 10]*

To address the lack of communication skills, most of the experts considered the hypothesis ‘behavioural practice/rehearsal during educational outreach visits’ to be feasible and potentially effective (246, 22.4%).

*“I think communication skills can be best obtained by training. It is difficult to get professionals together for training skills, so educational outreach visit seemed the most feasible. However, also in these trainings videos of ' good behaviour' can be used to discuss how you can communicate about certain diagnosis, treatments and so on.” [respondent 11].*

## Discussion

Our objective was to identify the relevant determinants of youth care guideline implementation and formulate feasible and potentially effective implementation hypotheses to address these determinants. The recruited experts identified determinants related to 1) engagement (i.e., guideline promotion, mandatory education, the presence of a motivated implementation leader, and management support) and 2) knowledge and skills (i.e., guideline knowledge and communication skills) as crucial for the implementation of guidelines.

To elicit changes in determinants relating to engagement, the BCTs practical support and action planning were predominantly considered feasible and potentially effective. Various implementation strategies aimed at developing stakeholder interrelationships were most frequently regarded as feasible and potentially effective in facilitating changes in practice. To elicit changes in professionals’ knowledge and skills, the BCTs providing instructions on guideline use and behavioural practice/rehearsal practice were predominantly considered feasible and potentially effective. The majority of experts deemed various strategies focused on training and educating stakeholders feasible and potentially effective in facilitating changes in practice.

In total, 46 different hypotheses were formulated to address determinants, ranging from 6 to 9 hypotheses per determinant. For each determinant, we provide an overview of the most feasible and potentially effective hypotheses (as evaluated by the experts) that organizations can use to develop a tailored implementation plan for youth care guidelines.

### Implementation determinants

#### Engagement

The implementation of youth care guidelines is a multifaceted process necessitating a systematic approach. It encompasses the dissemination, adoption, and sustained utilization of guidelines. As such, CPs should be aware of the existence and content of these guidelines, be motivated to apply them in practice, and continue their usage [64]. According to the experts in this study, determinants focusing on engagement (i.e. promoting guideline use, providing mandatory education, the presence of an implementation leader, and management support) were considered relevant to facilitate the implementation process. Engaging appropriate individuals to stimulate the implementation and use of guidelines and interventions is often overlooked during the implementation process [65]. However, involving all stakeholders, such as management, implementation leaders, and users, in the implementation of guidelines contributes to successful implementation [66]. Individuals who are more committed to their tasks and supported in their efforts contribute positively to the implementation process.

Mandatory education is categorised in the CFIR construct ‘Process—Engaging’. In this context, mandatory education serves to attract and involve professionals in the use of guidelines. Different theories exist regarding the effects of mandatory education. Some argue that trainees attending voluntary education programs display higher autonomous motivation, translating to genuine interest and personal commitment to guideline implementation [67, 68]. Contrary, others have shown increased motivation with mandatory education, suggesting that when education is mandatory, it must be important [69]. The enforced nature of the education might convey the significance of adherence to these guidelines, leading to increased motivation to implement them effectively.

The presence of a motivated implementation leader, identified as an important facilitator, aligns with findings from studies, particularly those focused on child abuse guidelines [33, 34, 36, 70, 71]. Implementation leaders play a pivotal role in facilitating successful guideline implementation by improving networks and communication, enabling access to experts, and lowering the threshold for professionals to seek assistance [70]. Their presence is also associated with professionals’ improved readiness to care for children and guideline implementation [72]. Furthermore, poor management support was considered a relevant barrier, which is in line with previous studies [34, 39].

#### Knowledge and skills

Consistent with prior research, knowledge about the use of the guideline was considered as a facilitator [33, 34, 73] while the lack of it was considered a barrier [37, 38, 43]. According to the behaviour framework by Cabana [74], increasing knowledge is expected to enhance positive attitudes toward the guidelines, ultimately contributing to the effective implementation of guidelines.

The experts considered poor communication skills to be a relevant barrier, aligning with findings from previous studies [35, 37, 40, 71, 75]. Effective communication skills are crucial in detecting psychosocial problems, as professionals' abilities and interviewing techniques are linked to parents' disclosure. However, professionals' communication skills often pose a significant challenge when discussing sensitive issues with patients or parents [76, 77].

Professionals’ perceived responsibility towards screening for psychosocial problems and their belief that using guidelines will result in positive outcomes were also considered important but more challenging to change in practice compared to factors like knowledge and the availability of resources. Attitudes and beliefs are shaped by past and present experiences [78] and once established, they are hard to change [79]. Crapazano [79] demonstrated that despite an increase in knowledge about alcohol and drug use, professionals' attitudes and beliefs about screening practices and interventions remained negative.

Consistent with the expert consensus study by Huijg [44], time availability is deemed important but challenging to alter in practice. Activities like screening for psychosocial problems, interprofessional collaboration, and family care are time-consuming. Despite the well-known time constraint in youth care, professionals require support from management and policymakers. Organizations can enhance time management through prioritization workshops, technology integration, purposeful scheduling, and team collaboration platforms. These strategies might empower professionals to navigate time constraints, enhancing overall productivity in the dynamic field of youth care [80, 81].

#### Contrary to previous literature

While the experts in the current study considered guideline promotion and mandatory education relevant for optimal implementation, they are rarely considered facilitators in other studies. This discrepancy could be explained by the fact that researchers often utilize frameworks [24, 33–35, 40, 71, 75] or questionnaires [39, 82] with preformulated determinants that may not specifically cover these determinants. Additionally, determinants are often identified from the perspective of professionals using the guidelines rather than those facilitating guideline implementation [34]. Consequently, determinants within the domain of the individual are more likely to be cited than determinants within the domain of the process, s the latter is more focused on the organizational level [30].

Contrary to our expectations, professionals' fear of false identification after screening for psychosocial problems was considered one of the least important determinants by the experts. This could be attributed to the fact that fear is a frequently cited barrier among mandated reporters of child abuse [73, 83–85]. However, in our study, questions were directed towards determinants of the implementation of youth care guidelines focusing on psychological, behavioural, and social problems in children and their families in general. These guidelines do not mandate professionals to report to authorities when they have doubts regarding a child’s development. In many countries, however, professionals are obligated by law to report any reasonable suspicion of child abuse. In child abuse, fear of false identification is therefore perceived as a major barrier.

### Implementation hypotheses

#### Engagement

To elicit change in engagement-related determinants, the BCTs practical support and action planning were considered the most feasible and potentially effective. There is growing interest in the use of action planning to bridge the gap between behavioural intentions and actual change. The development of an action plan can help initiate change by specifying when, where, and how to act [86, 87]. Effective action planning has the potential to enhacnce a positive workplace culture in which both management and professionals are actively engaged and take responsibility for guideline implementation and quality improvement [86, 88]. Practical, task–oriented support includes clarifying roles, providing resources to perform tasks, and monitoring implementation [89]. In Connell’s consensus study, 81% of the experts considered providing practical support to be linked to social influences – interpersonal processes that can cause individuals to change their thoughts, feelings, or behaviours [48].

Strategies concentrating on the development of stakeholder interrelationships, such as obtaining formal commitments, were regarded as the most feasible and potentially effective in inducing changes in engagement-related determinants. Obtaining formal commitments is recognized as an effective strategy, as indicated by Waltz's consensus study, to enhance the commitment, involvement, and accountability of managers and implementation leaders in the implementation process [46].

#### Knowledge and skills

To elicit change in professionals’ knowledge and skills, the experts deemed providing instructions on guideline use and engaging in the practice and rehearsal of the behaviour as the most feasible and potentially effective BCTs. These findings align with prior research investigating the connections between determinants of change and BCTs [48, 60].

Various strategies focusing on the training and education of stakeholders, including educational meetings, collaborative learning, and ongoing training, were considered feasible and potentially effective in facilitating change in practice, which is in line with previous research [46, 90, 91]. For example, collaborative learning, an educational approach involving groups of professionals working together to solve problems or create solutions, has shown positive outcomes in healthcare settings. A study on collaborative learning within youth-friendly health services demonstrated improvements in professionals' healthcare knowledge, use of evidence-based resources, empowerment to provide high-quality youth-friendly care, teamwork, and cooperation [90]. Ongoing training strategies (e.g. booster sessions and follow–up training) appear promising in maintaining acquired knowledge and skills [46, 92]. Concerning educational meetings, despite its significant potential in achieving success in knowledge translation and enhancing professionals' practice [93], a Cochrane review showed only small to moderate effects [94]. The review emphasizes that the effectiveness of these meetings is influenced by several factors. They prove most effective when utilizing a mixed interactive and didactic format; however, addressing highly complex behaviours may pose a challenge. The perceived seriousness of the targeted outcome affects effectiveness, with smaller impacts observed for outcomes seen as having less serious consequences for patients. Additionally, factors such as attendance rate, intensity, location, and initial compliance also play crucial roles in determining their effectiveness.

## Strengths and limitations

One of the strengths of this study is the use of a widely used theoretical method to guide the Delphi study in the formulation of implementation hypotheses. Additionally, we employed various theoretical frameworks to categorize implementation determinants and compile a set of effective and feasible BCTs and strategies. Assessing determinants, BCTs, and strategies with the support of theoretical frameworks helps ensure a theoretically informed approach rather than relying solely on pragmatic considerations. It is anticipated that applying systematic theory-based methods and frameworks will contribute to the long-term effectiveness of the implementation process [31].

Another strength of the study is that in addition to strategies, we used BCTs to hypothesise how determinants can be best addressed. Implementation research often provides details on the type of strategies to address determinants but fails to describe which techniques are applied to initiate behaviour change. The lack of theoretical rationale and detailed information on behaviour change processes not only limits the design and replication of implementation efforts but also makes it challenging to evaluate what actually contributes to their effectiveness [59, 95].

Several limitations should be acknowledged in this study. Firstly, the absence of professionals and policymakers among the experts may introduce bias in the results, as their perspectives on the significance of implementation determinants and effective strategies could differ from those of researchers and experts. [96].

Additionally, it's important to note that this Delphi study follows a modified version of the classical approach, which starts with closed-ended questions rather than open-ended ones. While this modification aims to provide a solid foundation by offering a set of determinants based on previous empirical research and reducing the workload for experts, it may lead to the omission of some crucial determinants, BCTs, and implementation strategies not included in the preselected list.

## Conclusion

This study offers valuable insights into youth care guideline implementation by systematically formulating hypotheses based on expert input. In contrast to studies primarily focusing on determinant-targeted strategies, we delve into specific techniques crucial for behavioural change. By integrating scientific literature with implementation experts' perspectives, our research provides a nuanced understanding of the complex processes vital for successful youth care guideline implementation. Experts identified determinants most relevant by experts for the implementation of youth care guidelines, encompassing engagement, knowledge, and skills. We presented an overview of corresponding implementation hypotheses to guide organizations, policymakers, and professionals in improving the implementation process and outcomes in youth care guidelines. Future research should move beyond superficial effectiveness assessments and delve into the intricacies of how and why implementation strategies lead to positive outcomes. This shift will contribute to a nuanced understanding of the complex dynamics in youth care guideline implementation. Evaluating techniques and processes provides valuable information to develop context-specific interventions, thereby strengthening the overall knowledge base in implementation science and the use of BCTs in healthcare. Additionally, involving stakeholders at all organizational levels during determinant identification and hypothesis formulation is crucial, recognizing that implementation is a multilevel process where each individual can contribute uniquely to improvement.

### Supplementary Information

Additional file 1. Recommendations for the Conducting and REporting of DElphi Studies (CREDES) checklist [53].

Additional file 2. Overview of participating experts (*n* = 14).

Additional file 3. Determinants’ level of importance and changeability as indicated by experts (*n* = 14).

Additional file 4. Summary of the results of Rounds 2 and 3 (*n* = 14).

## Data Availability

All data supporting the conclusions of this study are included in the paper and its additional files. Other supporting data are available at: 10.17026/dans-293-q3yx.

## Abbreviations

ERIC: Expert recommendations for implementing change
BCT: Behavioural change technique
TIBC: Theory–informed behaviour change
CFIR: Consolidated framework for implementation research
