# Supplementary material for: Expert consensus on multilevel implementation hypotheses to promote the uptake of youth care guidelines: a Delphi study
Source: Health Res Policy Syst. 2024 Aug 2;22:89. doi: 10.1186/s12961-024-01167-x (PMC11295487; doi:10.1186/s12961-024-01167-x)
Supplement: Supplementary file 2 — Additional file 2. Overview of participating experts (n = 14). [file 12961_2024_1167_MOESM2_ESM.docx]

| **Additional file 2.** Characteristics of experts (*n*=14) | | | |
| --- | --- | --- | --- |
| **Expert** | **Sex** | **Expertise** | **Experience in years** |
| 1 | Female | Research-based experience in implementation | 0-5 |
| 2 | Female | Research-based experience in implementation | 10-15 |
| 3 | Female | Research-based experience in implementation | 10-15 |
| 4 | Female | Research-based experience in implementation | 30-35 |
| 5 | Male | Research-based experience in implementation in youth care | 30-35 |
| 6 | Female | Practice-based experience in youth care guideline implementation | 10-15 |
| 7 | Female | Research-based experience in implementation | 10-15 |
| 8 | Female | Research-based experience in implementation in youth care | 10-15 |
| 9 | Male | Research-based experience in implementation in youth care | 25-30 |
| 10 | Female | Research-based experience in implementation in youth care | 15-20 |
| 11 | Female | Practice-based experience in youth care guideline implementation | 5-10 |
| 12 | Female | Research-based experience in implementation in youth care | 20-25 |
| 13 | Female | Research-based experience in implementation in youth care | 20-25 |
| 14 | Female | Practice-based experience in youth care guideline implementation | 15-20 |
