# Supplementary material for: Expert consensus on multilevel implementation hypotheses to promote the uptake of youth care guidelines: a Delphi study
Source: Health Res Policy Syst. 2024 Aug 2;22:89. doi: 10.1186/s12961-024-01167-x (PMC11295487; doi:10.1186/s12961-024-01167-x)
Supplement: Supplementary file 3 — Additional file 3. Determinants’ level of importance and changeability as indicated by experts (n = 14). [file 12961_2024_1167_MOESM3_ESM.docx]

| **Additional file 3.** Determinants’ level of importance and changeability as indicated by experts *(n=14)*. | | | | |
| --- | --- | --- | --- | --- |
| **Determinant** | **Importance** | | **Changeability** | |
|  | **Mn** | **Mdn** | **Mn** | **Mdn** |
| *Barriers* |  |  |  |  |
| Available Resources - Lack of equipment (materials, offices) | 6.21 | 6.50 | 4.00 | 4.00 |
| Available Resources - Lack of time | 7.86 | 8.00 | 2.64 | 2.00 |
| Compatibility - Poor congruence current workflow | 8.07 | 8.00 | 2.71 | 2.00 |
| Compatibility - High workload | 7.07 | 7.00 | 4.14 | 4.00 |
| Cosmopolitanism - Low confidence in follow-up care | 6.29 | 6.00 | 3.00 | 3.00 |
| Cosmopolitanism - Not along the same line regarding advice | 6.43 | 6.50 | 3.14 | 3.00 |
| Cosmopolitanism – Poor collaboration | 7.57 | 7.50 | 3.21 | 4.00 |
| Cosmopolitanism - Poor communication | 7.21 | 7.50 | 3.64 | 4.00 |
| Descriptive Norm - Poor guideline use by colleagues | 7.21 | 7.50 | 3.93 | 4.00 |
| Evidence Strength & Quality - Questions effectiveness of the guideline | 7.43 | 8.00 | 3.71 | 4.00 |
| Knowledge & Beliefs about the Innovation - Lack of knowledge regarding guideline use | 7.21 | 7.50 | 4.43 | 4.00 |
| Knowledge & Beliefs about the Innovation - Poor procedural clarity | 7.71 | 8.00 | 4.36 | 4.50 |
| **Leadership Engagement - Poor Management support** | **8.64** | **9.00** | **3.64** | **4.00** |
| Needs & Resources of those Served by the Organization - Poor client cooperation | 7.21 | 8.00 | 3.14 | 3.00 |
| **Other personal attributes - Lack of communication skills** | **8.43** | **9.00** | **3.79** | **4.00** |
| Professional’s Obligation - Not feeling responsible/motivated to use the guideline | 7.79 | 8.00 | 3.86 | 4.00 |
| Relationship Client - Fear of harming relationship with client | 7.57 | 8.00 | 2.86 | 2.50 |
| Self-efficacy - Difficult to perform dual role | 6.79 | 6.50 | 2.93 | 3.00 |
| Self-efficacy - Difficult to start conversation | 7.07 | 7.50 | 3.79 | 4.00 |
| Self-efficacy - Fear of false identification | 6.07 | 6.00 | 3.50 | 3.50 |
| *Facilitators* |  |  |  |  |
| Available Resources - Access to experts | 7.71 | 8.00 | 3.93 | 4.00 |
| Available Resources - Available equipment | 6.71 | 7.00 | 4.00 | 4.00 |
| Available Resources - Financial support | 6.29 | 7.00 | 2.93 | 3.00 |
| Available Resources - Time available | 8.21 | 8.50 | 2.57 | 2.00 |
| Compatibility - Congruence current workflow | 8.07 | 8.00 | 3.71 | 4.00 |
| Cosmopolitanism - Available for advice | 7.00 | 7.00 | 3.57 | 3.50 |
| Cosmopolitanism - Good collaboration | 7.64 | 7.50 | 2.79 | 3.00 |
| Cosmopolitanism - Good communication | 6.50 | 7.00 | 3.29 | 4.00 |
| Cosmopolitanism - Making an anonymous call to external organizations | 5.57 | 6.00 | 3.43 | 3.00 |
| Descriptive Norms - Use of guideline by colleagues | 7.86 | 8.00 | 3.57 | 4.00 |
| **Engaging – Guideline promotion** | **8.50** | **8.50** | **4.36** | **4.50** |
| Engaging - Direct/active involvement in implementation process | 7.71 | 8.00 | 4.07 | 4.00 |
| **Engaging - Mandatory education** | **8.07** | **8.50** | **4.43** | **4.50** |
| Engaging - Mandatory use of guideline | 6.71 | 7.00 | 3.57 | 4.00 |
| **Formally Appointed Implementation Leader - Presence of a motivated implementation leader** | **8.86** | **9.00** | **4.29** | **4.50** |
| **Knowledge & Beliefs about the Innovation - Knowledge regarding guideline use** | **8.29** | **8.50** | **3.79** | **4.00** |
| Knowledge & Beliefs about the Innovation - Procedural clarity | 8.00 | 8.00 | 4.21 | 4.00 |
| Leadership Engagement - Management support | 8.79 | 8.50 | 3.50 | 4.00 |
| Other personal attributes - Communication skills | 7.71 | 8.00 | 4.21 | 4.00 |
| Professional’s Obligation - Being motivated to use the guideline | 8.21 | 8.00 | 3.57 | 4.00 |
| Professional’s Obligation - Feeling responsible to use the guideline | 8.64 | 8.50 | 3.50 | 4.00 |
| Relationship Client - Building Relationship with client | 7.21 | 7.50 | 3.36 | 3.00 |
| Relationship Client - Transparency towards client | 7.50 | 7.00 | 3.79 | 4.00 |
| Relative Advantage - Believe in positive outcomes for the child | 8.64 | 9.00 | 3.36 | 4.00 |
| *Grand mean* | 7.51 |  | 3.60 |  |
| Mn=mean; Mdn=median; **bold**=determinants indicated by experts as most relevant for the implementation of youth care guidelines. | | | | |
