# Supplementary material for: Expert consensus on multilevel implementation hypotheses to promote the uptake of youth care guidelines: a Delphi study
Source: Health Res Policy Syst. 2024 Aug 2;22:89. doi: 10.1186/s12961-024-01167-x (PMC11295487; doi:10.1186/s12961-024-01167-x)
Supplement: Supplementary file 4 — Additional file 4. Summary of the results of Rounds 2 and 3 (n = 14). [file 12961_2024_1167_MOESM4_ESM.docx]

| **Additional file 4.** Formulated implementation hypotheses *(n=14)*. | | | | | |
| --- | --- | --- | --- | --- | --- |
|  | **Implementation hypotheses** | | **n (%)** | | **Category** |
|  | **Behavior change technique** | **Implementation strategy** | **R2** | **R3** | **Implementation strategy^a^** |
| **ENGAGEMENT** | *Promotion of guideline use* | |  |  |  |
|  | Habit formation | Use advisory boards and workgroups | 3 (21.4) | 6 (45.8) | Develop stakeholder interrelationships |
|  |  | Conduct educational meetings | 2 (14.3) | 2 (14.3) | Train and educate stakeholders |
|  |  | Involve executive boards | 1 (7.1) | - | Develop stakeholder interrelationships |
|  |  | Create a learning collaborative | 1 (7.1) | - | Train and educate stakeholders |
|  | Prompts/cues | Identify and prepare champions | 2 (14.3) | 2 (14.3) | Develop stakeholder interrelationships |
|  |  | Conduct educational meetings | 1 (7.1) | 1 (7.1) | Use evaluative and iterative strategies |
|  |  | Conduct local needs assessment | 2 (14.3) | 1 (7.1) | Train and educate stakeholders |
|  | Action planning | Create a learning collaborative | 1 (7.1) | 1 (7.1) | Train and educate stakeholders |
|  |  | Identify and prepare champions | 1 (7.1) | 1 (7.1) | Develop stakeholder interrelationships |
|  | *Mandatory education* | |  |  |  |
|  | Action planning | Create a learning collaborative | 2 (14.3) | 2 (14.3) | Train and educate stakeholders |
|  |  | Conduct local needs assessment | 3 (21.4) | 4 (28.5) | Use evaluative and iterative strategies |
|  |  | Use advisory boards and workgroups | 1 (7.1) | 2 (14.3) | Develop stakeholder interrelationships |
|  |  | Assess for readiness and | 1 (7.1) | 1 (7.1) | Use evaluative and iterative strategies |
|  |  | Involve executive boards | 1 (7.1) | - | Develop stakeholder interrelationships |
|  | Restructuring the physical environment | Create a learning collaborative | 2 (14.3) | 2 (14.3) | Train and educate stakeholders |
|  |  | Inform local opinion leaders | 1 (7.1) | 1 (7.1) | Develop stakeholder interrelationships |
|  |  | Use advisory boards and workgroups | 1 (7.1) | 1 (7.1) | Develop stakeholder interrelationships |
|  |  | Assess for readiness and identify barriers/facilitators | 1 (7.1) | 1 (7.1) | Use evaluative and iterative strategies |
|  | Prompt/cues | Use advisory boards and workgroups | 1 (7.1) | - | Develop stakeholder interrelationships |
|  | *Presence of an implementation leader* | |  |  |  |
|  | Social support (practical) | Provide ongoing consultation | 2 (14.3) | 3 (21.4) | Train and educate stakeholders |
|  |  | Identify and prepare champions | 1 (7.1) | 2 (14.3) | Develop stakeholder interrelationships |
|  |  | Organize clinician implementation team meetings | 1 (7.1) | - | Develop stakeholder interrelationships |
|  |  | Inform local opinion leaders | 1 (7.1) | 1 (7.1) | Develop stakeholder interrelationships |
|  | Social support (unspecified) | Inform local opinion leaders | 1 (7.1) | 1 (7.1) | Use evaluative and iterative strategies |
|  |  | Assess for readiness and identify barriers/facilitators | 2 (14.3) | 1 (7.1) | Develop stakeholder interrelationships |
|  |  | Identify and prepare champions | 1 (7.1) | 1 (7.1) | Develop stakeholder interrelationships |
|  | Social comparison | Recruit, designate and train for leadership | 1 (7.1) | 2 (14.3) | Develop stakeholder interrelationships |
|  |  | Develop a formal implementation blueprint | 1 (7.1) | - | Use evaluative and iterative strategies |
|  |  | Inform local opinion leaders | 1 (7.1) | - | Develop stakeholder interrelationships |
|  | Restructuring the social environment | Recruit, designate and train for leadership | 2 (14.3) | 3 (21.4) | Develop stakeholder interrelationships |
|  | *Poor management support* | |  |  |  |
|  | Social support (practical) | Conduct local consensus discussions | 3 (21.4) | 5 (35.7) | Develop stakeholder interrelationships |
|  |  | Obtain formal commitments | 2 (14.3) | 2 (14.3) | Develop stakeholder interrelationships |
|  |  | Involve executive boards | 1 (7.1) | 1 (7.1) | Develop stakeholder interrelationships |
|  |  | Recruit, designate and train for leadership | 1 (7.1) | 1 (7.1) | Develop stakeholder interrelationships |
|  | Restructuring the social environment | Develop a formal implementation blueprint | 1 (7.1) | 1 (7.1) | Use evaluative and iterative strategies |
|  |  | Obtain formal commitments | 1 (7.1) | 1 (7.1) | Develop stakeholder interrelationships |
|  | Social comparison | Involve executive boards | 1 (7.1) | 1 (7.1) | Develop stakeholder interrelationships |
|  |  | Identify and prepare champions | 1 (7.1) | 1 (7.1) | Develop stakeholder interrelationships |
|  | Social support (unspecified) | Develop a formal implementation blueprint | 1 (7.1) | - | Use evaluative and iterative strategies |
|  | Monitoring behavior without feedback | Conduct local consensus discussions | 1 (7.1) | - | Develop stakeholder interrelationships |
|  | Social rewards | Alter incentive/allowance structures | 1 (7.1) | 1 (7.1) | Utilize financial strategies |
| **KNOWLEDGE & SKILLS** | *Knowledge regarding the use of the guideline* | |  |  |  |
|  | Feedback on behavior | Conduct educational meetings | 2 (14.3) | 1 (7.1) | Train and educate stakeholders |
|  |  | Create a learning collaborative | 2 (14.3) | 3 (21.4) | Train and educate stakeholders |
|  |  | Conduct educational outreach visits | 1 (7.1) | - | Train and educate stakeholders |
|  | Instruction on how to perform a behavior | Conduct educational meetings | 3 (21.4) | 4 (28.5) | Train and educate stakeholders |
|  |  | Create a learning collaborative | 1 (7.1) | 2 (14.3) | Develop stakeholder interrelationships |
|  | Information about antecedents | Distribute educational materials | 1 (7.1) | 1 (7.1) | Train and educate stakeholders |
|  |  | Identify and prepare champions | 1 (7.1) | 1 (7.1) | Train and educate stakeholders |
|  |  | Conduct educational meetings | 1 (7.1) | - | Develop stakeholder interrelationships |
|  | Information about health consequences | Conduct educational meetings | 1 (7.1) | 1 (7.1) | Train and educate stakeholders |
|  |  | Conduct educational outreach visits | 1 (7.1) | 1 (7.1) | Train and educate stakeholders |
|  | *Lack of communication skills* | |  |  |  |
|  | Behavioral practice/rehearsal | Conduct educational outreach visits | 3 (21.4) | 2 (14.3) | Train and educate stakeholders |
|  |  | Conduct ongoing training | 4 (28.5) | 8 (57.1) | Train and educate stakeholders |
|  |  | Create a learning collaborative | 1 (7.1) | 1 (7.1) | Train and educate stakeholders |
|  |  | Conduct educational meetings | 1 (7.1) | - | Train and educate stakeholders |
|  |  | Assess for readiness and identify barriers/facilitators | 1 (7.1) | 1 (7.1) | Use evaluative and iterative strategies |
|  | Demonstration of the behavior | Conduct ongoing training | 1 (7.1) | 1 (7.1) | Train and educate stakeholders |
|  |  | Conduct educational meetings | 1 (7.1) | 1 (7.1) | Train and educate stakeholders |
|  | Problem solving | Conduct ongoing training | 1 (7.1) | - | Train and educate stakeholders |
|  | Graded tasks | Create a learning collaborative | 1 (7.1) | - | Train and educate stakeholders |
| R1=Round1; R2=Round 2.  ^a^ Categories based by Waltz et al., 2015. | | | | |  |
